# Self-Reported Well-Being and Health Among Deaf and Hard-of-Hearing Adolescents in Mainstream Schools: A Swedish School Survey Study

**DOI:** 10.3390/children13020308

**Published:** 2026-02-23

**Authors:** Sylvia Olsson, Carina Loeb

**Affiliations:** 1 Division of Social Work, Department of Social Sciences and Humanities, Mälardalen University, SE-631 05 Eskilstuna, Sweden; 2 Division of Psychology, Department of Social Sciences and Humanities, Mälardalen University, SE-631 05 Eskilstuna, Sweden; carina.loeb@mdu.se

**Keywords:** deaf and hard-of-hearing adolescents, well-being, mental health complaints, somatic complaints, teacher support

## Abstract

**Highlights:**

**What are the main findings?**

- Deaf and hard-of-hearing (DHH) adolescents in mainstream schools report poorer well-being and higher levels of somatic and mental health complaints than hearing peers.
- DHH adolescents with additional disabilities consistently show the poorest outcomes, including the lowest levels of perceived teacher support.

**What are the implications of the main findings?**

- Teacher support and communication-sensitive teaching practices are critical targets for promoting well-being among DHH adolescents in mainstream schools.
- Early identification of somatic and mental health complaints in school settings may help prevent long-term health inequalities among adolescents with hearing loss.

**Abstract:**

Background: Deaf and hard-of-hearing (DHH) adolescents in mainstream schools may face communication barriers and social challenges that can affect their well-being and health. However, population-based knowledge based on adolescents’ own reports—particularly including those with additional disabilities—remains limited. The aim of this study was to assess self-reported well-being, mental health complaints, somatic complaints, and perceived teacher support among DHH adolescents in Swedish mainstream schools and to compare these outcomes with those of hearing adolescents and DHH adolescents with additional disabilities. Methods: This cross-sectional study was based on data from the Swedish school survey Liv och Hälsa Ung (Life and Health of Young People). The sample comprised 5923 adolescents aged 13–18 years attending grades 7 and 9 in compulsory school and year 2 in upper-secondary school. Outcomes included well-being (WHO-5 or a single-item measure for grade 7), mental health complaints, somatic complaints, and perceived teacher support. Group differences by hearing status, additional disability, gender, and school level were examined using analysis of variance (ANOVA). Results: Hearing adolescents reported higher well-being, fewer somatic complaints, fewer mental health complaints, and higher perceived teacher support compared with DHH adolescents. DHH adolescents with additional disabilities consistently reported the poorest outcomes across all domains. For example, perceived teacher support was significantly lower among DHH adolescents with additional disabilities (M = 3.66, 95% CI [3.54–3.78]) compared with hearing adolescents (M = 4.01, 95% CI [3.99–4.03]). Across all groups, girls, particularly those with disabilities, reported poorer well-being and higher levels of somatic and mental health complaints than boys. Conclusions: The findings highlight substantial health disparities among adolescents with hearing loss in mainstream schools, especially among those with additional disabilities. Perceived teacher support emerged as an important contextual factor and may represent a key target for school-based interventions aimed at promoting well-being and mental health among DHH adolescents.

## 1. Introduction

This study focuses on self-reported well-being, mental health complaints, and somatic complaints among deaf and hard-of-hearing (DHH) adolescents in mainstream schools. The term DHH refers to individuals with partial or total hearing loss, ranging from slight to profound, and encompasses a heterogeneous group with varying communication needs and educational experiences [1,2].

In many countries, including Sweden, the majority of DHH adolescents attend mainstream schools, often in their local communities [3,4,5,6]. While mainstream schooling is intended to promote inclusion, DHH adolescents may face communication barriers, limited access to social interaction, and challenges in classroom participation. These challenges can increase the risk of social exclusion, bullying, and stress, which in turn may affect adolescents’ well-being and health [7,8,9,10]. Educational placement and the quality of support provided in school have been shown to play an important role in the everyday experiences of DHH adolescents [11,12]. In mainstream classrooms, support is often embedded within regular teaching practices, and the extent to which teaching is adapted to meet the communication needs of DHH students varies widely. Large class sizes, playgrounds, suboptimal sound environments, and limited teacher awareness of hearing-related needs may further contribute to increased listening effort and fatigue for DHH adolescents [9,13,14].

Playground behavior studies in mainstream settings demonstrate that DHH children engage less in peer interactions compared with hearing peers, underscoring how communication barriers can extend beyond classroom instruction to social contexts [9]. Despite the high proportion of DHH adolescents in mainstream schools, there is limited population-based knowledge based on adolescents’ own reports regarding their well-being, mental health complaints, somatic complaints, and perceived teacher support. This gap is particularly evident for DHH adolescents with additional disabilities, who may face compounded challenges in the school environment [15,16].

### 1.1. Well-Being, Mental Health Complaints, and Somatic Complaints Among DHH Adolescents

Well-being, mental health complaints, and somatic complaints are closely related yet conceptually distinct dimensions of adolescents’ health and development. Well-being is commonly understood as a broader, positive conception of mental health that encompasses individuals’ subjective evaluations of their functioning, life satisfaction, and vitality, rather than merely the absence of illness or symptoms [2,17]. In contrast, mental health complaints refer to specific psychological symptoms such as stress, anxiety, low mood, or irritability, which may vary in intensity and duration and do not necessarily indicate a clinical diagnosis [2,17]. Somatic complaints, including headache, stomach pain, sleep difficulties, and fatigue, are common during adolescence and often co-occur with mental health complaints, reflecting the interplay between psychological strain and physical symptoms during this developmental period [18].

While these dimensions of health are conceptually distinct, they are also interrelated. Well-being is commonly conceptualized as a positive dimension of mental health reflecting vitality, life satisfaction, and functioning, whereas mental health complaints refer to specific psychological symptoms such as anxiety, stress, or low mood [17,18]. Somatic complaints, including headache, stomach pain, and sleep difficulties, frequently co-occur with psychological distress during adolescence, reflecting the close interplay between emotional strain and physical symptoms [18].

Lower levels of subjective well-being may therefore co-occur with elevated mental health complaints, which in turn may be reflected in increased somatic symptoms. At the same time, each dimension captures unique information, as adolescents may report relatively high well-being despite experiencing specific symptoms, or vice versa. Given both their conceptual differences and empirical interrelationships, a multidimensional approach is necessary to capture the complexity of adolescents’ health and well-being [19]. Assessing well-being alongside mental and somatic complaints allows for a more nuanced understanding of how different dimensions of health may vary among deaf and hard-of-hearing (DHH) adolescents and in relation to school-related factors such as perceived teacher support.

Adolescence is a critical developmental period during which patterns of well-being and health are established. A decline in well-being and an increase in mental and somatic complaints during adolescence constitute a major public health concern in many countries, including Sweden [19,20]. Population-based surveys indicate that mental health complaints and somatic symptoms increase with age during adolescence. For example, in a large repeated cross-sectional study from the Netherlands, emotional symptoms showed a small increase between 2009 and 2013, and psychosomatic complaints increased steadily between 2005 and 2013, while life satisfaction declined to levels below the 2005 baseline by 2013 and remained lower in 2017 [21]. These trends were also more pronounced among girls than boys in several high-income countries. Such patterns are likely driven by multiple interacting mechanisms, including heightened academic and social demands, puberty-related changes, increased stress exposure, and gendered norms regarding the expression and reporting of psychological and physical symptoms. Similar age- and gender-related patterns have been reported in other European countries, strengthening the generalizability of these trends across high-income contexts.

From an ecological perspective [22], adolescent development and health—particularly the experiences of deaf and hard-of-hearing (DHH) adolescents—are shaped by interactions across multiple environmental contexts. During adolescence, school, peer, and family settings are especially influential for emotional and social development. Supportive relationships within these contexts—such as responsive teachers and positive family relations—are associated with higher well-being, whereas stressors such as academic pressure, social exclusion, or negative school experiences are linked to poorer mental health outcomes. For DHH adolescents, communication barriers and varying levels of school support may further shape how these ecological contexts influence well-being and health.

At the individual level, characteristics such as hearing status, gender, age, and the presence of additional disabilities may influence health and well-being. At the microsystem level, everyday interactions with teachers, peers, and family constitute central contexts in which DHH adolescents experience inclusion, participation, and support in school. In particular, the quality of teacher–student relationships and perceived teacher support constitute key mechanisms through which the school microsystem may promote or hinder DHH adolescents’ well-being and mental health. The mesosystem highlights the interconnections between home and school; for DHH adolescents, limited coordination between schools, health services, and families may reduce the effectiveness of support and exacerbate stress related to communication barriers and participation in school. The exosystem and macrosystem encompass broader educational policies, societal attitudes toward disability, and the organization of inclusive education in Sweden. This ecological framing underscores that disparities in well-being, mental health complaints, and somatic complaints among DHH adolescents should be understood in relation to both individual circumstances and the social and educational environments in which they are embedded [23].

Adolescents with disabilities report poorer health compared with their non-disabled peers, not necessarily due to the impairment itself but as a result of environmental and social conditions [24]. In adolescence, hearing loss has been associated with lower health-related quality of life, underscoring the importance of examining subjective well-being among DHH youth. Recent research shows that adolescents with bilateral permanent hearing loss report significantly lower health-related quality of life compared with their hearing peers, reflecting broader challenges in emotional and social functioning that go beyond academic outcomes [25]. Previous research has demonstrated that DHH adolescents are at increased risk of mental health complaints and somatic problems compared with hearing adolescents [26,27,28]. A systematic review and meta-analysis found that children and adolescents with hearing impairment show elevated emotional and behavioral difficulties, including internalizing problems such as anxiety and depression, compared with hearing peers [29]. Studies have also reported lower levels of well-being and quality of life among DHH adolescents, particularly among those with additional disabilities [17,18]. More recent population-based research has similarly shown reduced health-related quality of life among children and adolescents with hearing loss, reinforcing the importance of examining subjective well-being in this group [25].

Several factors may contribute to these disparities. DHH adolescents often face challenges related to communication, language development, and social interaction, which may increase stress and listening effort in everyday school situations [30]. Prolonged listening effort and communication barriers have been linked to fatigue, stress-related somatic complaints, and poorer mental health [15,16,31]. Together, these findings highlight the importance of examining well-being, mental health complaints, and somatic complaints simultaneously in studies of DHH adolescents’ school experiences.

### 1.2. School Context and Health Among DHH Adolescents in Mainstream Schools

In mainstream schools educating deaf and hard-of-hearing (DHH) adolescents, teachers may face challenges in meeting diverse communication needs, partly due to the wide variation in students’ hearing loss and communication skills [32,33,34]. Communication barriers can limit social interaction with hearing peers and teachers, which may increase the risk of social exclusion in the school environment [7,8,10,33,34]. Empirical research indicates that difficulties with peer relationships and social inclusion are common among DHH adolescents in mainstream settings. Compared with hearing peers, DHH adolescents report higher levels of peer problems, including lower friendship quality and more frequent experiences of social isolation or exclusion, particularly in contexts where communication barriers are prominent. Qualitative and mixed-methods studies further highlight that many DHH students describe feelings of being socially marginalized in mainstream classrooms, emphasizing that reduced opportunities for spontaneous interaction are central to these experiences [7,8,10,33,34].

For some DHH adolescents, social exclusion in mainstream schools is a recurring experience. Such exclusion may arise from social stigma and difficulties in participating in spontaneous classroom interactions and informal peer communication [35,36]. Adolescents themselves report that limited awareness and stigma surrounding hearing loss contribute to stress and social tension in school environments, suggesting that well-being among DHH students cannot be fully understood without considering peer attitudes and knowledge [35]. Limited access to effective communication and supplementary learning opportunities may further negatively affect students’ well-being and mental health. Previous studies have shown that DHH adolescents may feel different from their peers and experience exclusion related to the use of assistive hearing technology [5,36]. Recent observational research in mainstream school settings also demonstrates that DHH children engage less in spontaneous peer interactions during unstructured activities such as playground time, indicating that challenges to social inclusion extend beyond the classroom [9]. More broadly, social relationships and opportunities for communication with peers and adults are central to adolescents’ sense of inclusion and well-being [37,38]. Within the school context, survey-based research has shown that communication and social experiences are associated with school satisfaction, quality of life, and mental health among DHH adolescents [29].

Somatic complaints, such as headache, stomach pain, back pain, fatigue, dizziness, and stress, are also common among DHH adolescents in school settings [2]. Stress related to communication difficulties and sustained listening effort may contribute to fatigue and stress-related somatic complaints [13,14,39]. During adolescence, somatic complaints are closely associated with mental health complaints, including anxiety and depressive symptoms [18,40].

From an ecological perspective, these patterns illustrate how interactions within the school microsystem (classroom practices, peer relations, and teacher–student relationships) shape DHH adolescents’ everyday experiences of participation and well-being. Limited alignment between the school, family, and health services at the mesosystem level may further exacerbate vulnerabilities related to communication barriers, stress, and exclusion. At the broader exosystem and macrosystem levels, educational policies, resource allocation, and societal attitudes toward disability influence the extent to which mainstream schools are able to provide inclusive and supportive environments for DHH students.

Although school is a key developmental context for all children and adolescents, there remains limited population-based knowledge based on adolescents’ own reports regarding well-being, mental health complaints, and somatic complaints among DHH adolescents attending mainstream schools in Sweden. Much of the existing research is dated or based on small samples [41,42,43,44]. Moreover, few studies have examined how school-related factors, such as teacher support, are associated with health outcomes across gender, school level, and disability status among DHH adolescents.

The aim of this study was to assess self-reported well-being, mental health complaints, somatic complaints, and perceived teacher support among deaf and hard-of-hearing (DHH) adolescents in Swedish mainstream schools and to compare these outcomes with those of hearing adolescents and DHH adolescents with additional disabilities.

Based on previous research demonstrating elevated mental health complaints, somatic problems, and lower health-related quality of life among DHH adolescents, particularly among those with additional disabilities [15,16,24,25,26,27,28], as well as established gender differences in adolescent mental and somatic health [21,22], we propose the following hypotheses:

**Hypothesis 1.**  *Hearing adolescents report higher well-being, fewer mental health complaints, and fewer somatic complaints compared with DHH adolescents, while DHH adolescents with additional disabilities report the poorest outcomes.*

**Hypothesis 2.**  *Hearing adolescents report higher perceived teacher support compared with DHH adolescents, while DHH adolescents with additional disabilities report the lowest levels of perceived teacher support.*

**Hypothesis 3.**  *Girls report poorer well-being and higher levels of mental health complaints and somatic complaints than boys across all groups, with more pronounced disparities among adolescents with disabilities.*

## 2. Materials and Methods

### 2.1. Research Design

This study has a cross-sectional design based on data derived from the web-based survey Liv och Hälsa Ung (Life and Health of Young People), developed and administered by Region Örebro County, Örebro, Sweden, in collaboration with the municipalities in the region. The survey is developed by the working group within the cooperating county councils/regions, and data has been collected every three years since 2005. For this study, data from 2017 was used, as this was the most recent survey wave that included all measures required for the present analyses across the relevant school levels. The questions are intended for gathering knowledge about young people’s health, living conditions, and living habits. Results for the questions in the survey have been published in earlier studies for similar groups of participants [16,45,46,47,48].

### 2.2. Participants

The sample comprised adolescents aged 13–18 years attending grades 7 and 9 in compulsory schools and year 2 in upper-secondary schools in all municipalities of Örebro County, Sweden (approximately 300,000 inhabitants). All eligible students in these grades were invited to participate in the survey, and participation was voluntary.

After removing 86 adolescents who neither identified themselves as girls nor boys from the sample due to the small size of this group, the sample consisted of 5923 adolescents. The completion rate was 70–85%, with the highest proportion of respondents in year 7 and slightly lower among the older students. The majority (90%) had no reported disability. In total, 4 percent of the participants had a hearing disability, and 4 percent reported that they had a hearing disability and at least one additional disability. A total of 116 adolescents did not answer the question about disability, and nine did not answer the question about gender. See Table 1 for the frequencies (and percentages) of different types of participants based on the type of school, gender and disability. For the DHH participants in the present study, adolescents self-reported their hearing disability as slight, moderate, severe, or profound hearing loss. All of the participants have different needs for communication access depending on their individual needs and contexts, but the schools in Sweden must support students with all disabilities. To do that, schools must offer teachers with the right skills, who have the ability to meet the students’ needs to achieve the educational goals on their own conditions. This means that, in Sweden, DHH people have the right to use Swedish Sign Language (SSL) in the educational setting and are given the opportunity to learn, develop, and use it as they wish. In all educational settings, they depend on hearing loops and FM systems, which all schools in Sweden have to provide.

### 2.3. Procedure

Teachers of grades 7 and 9 at compulsory schools and year 2 at upper-secondary schools in all municipalities of the county of Örebro in Sweden distributed a letter to each adolescent containing information about a web-based survey and login details with a unique code. The adolescents were informed that participation was voluntary, and they answered the questionnaire individually and confidentially in the classroom. The adolescents were considered to be fully competent to give their consent and answer the survey [47].

### 2.4. Material

In the current study, the demographic characteristics of gender, year in school, and disability were used as independent measures. The following outcomes (dependent measures) were analyzed: (a) well-being (WHO-5 or general well-being), (b) mental health complaints, (c) somatic complaints, and (d) perceived teacher support.

Demographic characteristics. The demographic characteristics were measured as follows: gender, 1 = girl, 2 = boy; year in school, 1 = 7th grade in compulsory schools, 2 = 9th grade in compulsory schools, 3 = year 2 in upper-secondary schools; and disability, 0 = hearing adolescents, 1 = DHH adolescents, 2 = DHH adolescents with additional disability. The following options were given for additional disability: vision impairment that cannot be corrected with glasses or contact lenses, mobility impairment, reading difficulties, writing difficulties, dyslexia, ADHD or ADD, and other disabilities. Hearing loss status was based on students’ self-reports of having a hearing impairment. The survey did not include information on the degree or type of hearing loss, communication modality, use of hearing technology, or the functional impact of the impairment in everyday school situations. Consequently, the DHH group should be considered heterogeneous with respect to severity and functional consequences of hearing loss.

Well-being (WHO-5). For adolescents in the 9th grade and year 2 in upper-secondary schools, well-being was measured with the WHO-5 [49] which is among the most widely used questionnaires assessing subjective psychological well-being and has been translated into more than 30 languages. It consists of 5 items and has been found to have adequate validity, both as a screening tool for depression and as an outcome measure in clinical trials, and it has been successfully applied across a wide range of fields [47]. A sample item is “I have felt cheerful and in good spirits.” The items were rated based on the respondents’ experiences during the last two weeks, and a 6-point response scale ranging from 0 (at no time) to 5 (all of the time) was used. Cronbach’s alpha was 0.86.

General well-being. In order to keep the number of questions down for the younger adolescents in 7th grade, a single item was used to measure general well-being instead of the WHO-5: “How do you feel in general?” A 5-point response scale ranging from 1 (very bad) to 5 (very good) was used.

Somatic problems. Nine items were used to measure the following somatic problems during the last three months: headache, migraine, stomachache, pain in shoulders/neck, pain in the back/hips, pain in hands/knees/legs/feet, tinnitus, difficulty falling asleep and restless sleep. The items were rated on a 5-point scale ranging from 1 (never) to 5 (always). Cronbach’s alpha was 0.81.

Mental health complaints. In this study, mental health complaints refer to self-reported symptoms such as stress, worry, low mood, and irritability, based on survey responses. Mental health complaints were measured using five indicators assessed by the question “During the last three months, how often have you felt…?”: stressed, anxious/worried, sad/depressed, feeling in control (reverse coded), and irritated. Responses were rated on a 5-point scale ranging from 1 (never) to 5 (always). The items were averaged to form an index, which demonstrated acceptable internal consistency (Cronbach’s α = 0.72).

Perceived teacher support. Perceived teacher support was measured using a four-item scale (index) capturing students’ general perceptions of support from their teachers. The items were: (a) “My teachers make me believe in myself and my schoolwork”; (b) “My teachers help me with my schoolwork when I need it”; (c) “My teachers expect me to reach the goals in all subjects”; and (d) “I feel that my teachers listen to my opinions.” The items capture both emotional/relational and academic aspects of teacher support.

Responses were given on a 5-point Likert scale ranging from 1 (strongly disagree) to 5 (strongly agree), with higher scores indicating higher perceived teacher support. The scale demonstrated good internal consistency (Cronbach’s α = 0.87).

This measure captures students’ perceived general (emotional/relational and academic) teacher support and does not include communication-specific accommodations for DHH students, such as the use of FM systems, captioning, classroom acoustics, or visual positioning in the classroom. We therefore interpret teacher support in this study as students’ general perception of supportive teacher–student relationships rather than as a measure of specialized pedagogical or technical support related to hearing loss.

The items used to construct these measures were drawn from the Swedish school survey Liv och Hälsa Ung and have been used in previous population-based studies of adolescent health in Sweden. Although these scales are not standardized clinical instruments, they are widely used in Swedish public health monitoring and have demonstrated acceptable internal consistency in prior research. For the present study, internal consistency was assessed using Cronbach’s alpha, which ranged from 0.72 to 0.87 across scales, indicating acceptable to good reliability. Given the secondary nature of the survey data and the fixed item structure of the questionnaire, confirmatory factor analysis (CFA) was not conducted; instead, internal consistency was evaluated using Cronbach’s alpha, which is commonly applied in population-based school surveys.

### 2.5. Data Analysis

Analyses were performed using the Statistical Package for the Social Sciences (SPSS), version 28.0. Descriptive statistics were used to describe the characteristics of the study population and its subgroups; all percentages presented are based on valid responses, with missing values excluded.

Pearson correlation analyses were conducted to examine associations between well-being, mental health complaints, and somatic complaints. To examine group differences by hearing status, additional disability, gender, and school level, analyses of variance (ANOVAs) were performed. Multivariate ANOVAs were used when outcomes were analyzed simultaneously (Hypotheses 1 and 3), whereas one-way ANOVAs with Tukey post hoc tests were used to examine differences in perceived teacher support (Hypothesis 2). Prior to conducting the inferential analyses, the assumptions underlying Pearson correlations, ANOVA, and MANOVA were examined. Normality was assessed through inspection of skewness and kurtosis values as well as visual inspection of residual plots. Homogeneity of variance was evaluated using Levene’s test for the ANOVA models. For multivariate analyses, multivariate normality was evaluated through inspection of residual distributions, and Box’s M test was used to assess the homogeneity of covariance matrices. Given the large sample size, minor deviations from normality were considered acceptable, as parametric tests are generally robust under such conditions. Overall, the assumptions were deemed sufficiently satisfied for the planned analyses. To reduce the risk of Type I error associated with multiple comparisons, multivariate analyses (MANOVAs) were conducted when several related dependent variables were examined simultaneously. For post hoc pairwise comparisons following significant ANOVAs, Tukey’s HSD test was applied, which adjusts for multiple comparisons. No additional correction (e.g., Bonferroni adjustment) was applied, as the analyses were theory-driven and based on predefined hypotheses. A significance level of *p* < 0.05 was applied to all statistical tests.

## 3. Results

The overall results showed that higher levels of somatic complaints were associated with lower general well-being (r(*N* = 5882) = −0.50, *p* < 0.001) and higher levels of mental health complaints (r(*N* = 5844) = 0.64, *p* < 0.001). This pattern remained among DHH adolescents (see Table 2).

Gender differences were observed across all health outcomes. Girls reported lower well-being (M = 2.60, SD = 1.02) than boys (M = 3.35, SD = 0.98; F(1, 3708) = 50.94, *p* < 0.001, partial η^2^ = 0.013), higher levels of somatic complaints (M = 2.24, SD = 0.66) than boys (M = 1.85, SD = 0.61; F(1, 5766) = 90.96, *p* < 0.001, partial η^2^ = 0.015), and higher levels of mental health complaints (M = 2.96, SD = 0.86) than boys (M = 2.21, SD = 0.79; F(1, 5738) = 185.62, *p* < 0.001, partial η^2^ = 0.025).

Hypothesis 1 was supported for all three health outcomes. Disability status had a significant main effect on well-being (F(2, 3708) = 37.73, *p* < 0.001, partial η^2^ = 0.020). As shown in Table 3, hearing adolescents reported the highest levels of well-being (M = 3.01, SD = 0.87), followed by DHH adolescents (M = 2.80, SD = 1.04), while DHH adolescents with additional disabilities reported the lowest well-being (M = 2.37, SD = 1.09). Bonferroni-adjusted post hoc comparisons indicated that all three groups differed significantly from one another (*p* < 0.05).

Disability status also had a significant main effect on somatic complaints (F(2, 5766) = 120.58, *p* < 0.001, partial η^2^ = 0.040). Hearing adolescents reported the lowest levels of somatic complaints (M = 2.01, SD = 0.61), followed by DHH adolescents (M = 2.31, SD = 0.71), whereas DHH adolescents with additional disabilities reported the highest levels (M = 2.62, SD = 0.69). All pairwise differences were significant (Bonferroni-adjusted, *p* < 0.001).

Finally, disability status had a significant main effect on mental health complaints (F(2, 5738) = 48.35, *p* < 0.001, partial η^2^ = 0.025). Hearing adolescents reported the lowest levels of mental health complaints (M = 2.55, SD = 0.81), compared with DHH adolescents (M = 2.79, SD = 0.83), while DHH adolescents with additional disabilities reported the highest levels (M = 3.06, SD = 0.86). All pairwise differences were significant (Bonferroni-adjusted, *p* < 0.001). [Table 3].

Hypothesis 2 examined group differences in perceived teacher support. A one-way ANOVA showed a significant main effect of disability status on perceived teacher support (F(2, 5693) = 25.14, *p* < 0.001, partial η^2^ = 0.003). Estimated marginal means indicated that perceived teacher support was highest among hearing adolescents (M = 4.01, 95% CI [3.99–4.03]), lower among DHH adolescents (M = 3.89, 95% CI [3.83–3.95]), and lowest among DHH adolescents with additional disabilities (M = 3.66, 95% CI [3.54–3.78]). All pairwise comparisons were significant (Bonferroni-adjusted, *p* < 0.05).

Hypothesis 3, examining differences across gender, disability status, and year in school, was partially supported. A significant three-way interaction effect between gender, disability status, and year in school was found for somatic complaints (F(4, 5766) = 3.77, *p* = 0.005, partial η^2^ = 0.003). As shown in Table 4, DHH girls with additional disabilities in 7th grade reported the highest levels of somatic complaints (M = 2.91, SD = 0.55), whereas hearing boys in 7th grade reported the lowest levels (M = 1.76, SD = 0.54).

A significant three-way interaction effect was also found for mental health complaints (F(4, 5738) = 2.89, *p* = 0.021, partial η^2^ = 0.002). In line with Hypothesis 3, DHH girls with additional disabilities in year 2 of upper-secondary school reported the highest levels of mental health complaints (M = 3.51, SD = 0.89), whereas hearing boys in 7th grade reported the lowest levels (M = 1.98, SD = 0.70).

## 4. Discussion

This study examined self-reported well-being, somatic complaints, mental health complaints, and perceived teacher support among deaf and hard-of-hearing (DHH) adolescents in mainstream schools. The findings reveal clear and consistent disparities between hearing adolescents, DHH adolescents, and DHH adolescents with additional disabilities. Across all outcomes, adolescents with hearing loss, particularly those with additional disabilities, reported poorer well-being and higher levels of somatic and mental health complaints compared with their hearing peers. Interpreted through an ecological lens, these disparities reflect interactions between individual characteristics (e.g., hearing status, gender, additional disabilities) and multiple layers of the school and social environment.

### 4.1. Well-Being, Somatic Complaints, and Mental Health Complaints Among DHH Adolescents

In line with Hypothesis 1, moderate to strong associations were observed between well-being, somatic complaints, and mental health complaints. Adolescents reporting lower well-being also reported higher levels of somatic and mental health complaints, a pattern evident both in the total sample and among DHH adolescents specifically. These findings are consistent with previous research demonstrating poorer health outcomes among DHH adolescents, but the present study extends this literature by simultaneously examining multiple dimensions of health in a large population-based sample rather than relying on single-outcome indicators [26,27,28,49,50,51,52].

For example, Hintermair [29] reported lower health-related quality of life and greater psychosocial difficulties among DHH students in mainstream schools, particularly in relation to peer relationships and school participation. These findings suggest that barriers to social participation within mainstream educational settings may constitute an important mechanism underlying disparities in well-being and health among DHH adolescents. By integrating well-being, mental health complaints, and somatic complaints within the same analytical framework, the present study provides a more comprehensive assessment of health disparities across hearing status and additional disability.

From an ecological perspective, these patterns can be understood as reflecting interactions between the microsystem (classroom practices, peer relations, and teacher–student relationships) and the individual level (hearing status, additional disabilities, and stress sensitivity). Communication barriers, increased listening effort, and reduced opportunities for spontaneous interaction may contribute to sustained strain in everyday school situations, which may in turn be related to poorer physical and psychological well-being [26,28]. At the mesosystem level, limited alignment between home, health services, and school support may further exacerbate these vulnerabilities. At broader levels, educational policies, resource allocation, and societal attitudes toward disability shape the extent to which mainstream schools can provide supportive environments for DHH students.

### 4.2. Perceived Teacher Support in Mainstream Schools

Hypothesis 2 was supported, as clear group differences were found in perceived teacher support. Hearing adolescents consistently described the most supportive teacher relationships, while DHH adolescents reported lower levels of support, and those with additional disabilities perceived the least support overall. This pattern suggests that teacher–student relationships may be experienced differently depending on both hearing status and the presence of additional disabilities.

These findings highlight the central role of teachers in shaping adolescents’ school experiences, particularly for students with hearing loss. It should be noted that in this study, perceived teacher support reflects students’ general perceptions of emotional, relational, and academic support from their teachers, rather than communication-specific accommodations. Thus, the observed group differences primarily capture variations in students’ perceived relational support within everyday classroom interactions, rather than differences in specialized pedagogical or technical provisions.

Previous research has linked lower perceived teacher support to poorer mental health outcomes among adolescents more generally, typically measured through self-report scales of teacher–student relationships and classroom climate. In studies of DHH students, Hintermair [29] found that perceived classroom participation and teacher responsiveness were significantly associated with higher health-related quality of life. The present study adds to this literature by demonstrating, in a large population-based sample, that perceived teacher support is lowest among DHH adolescents with additional disabilities. However, future research would benefit from incorporating measures that also capture communication-specific accommodations (e.g., classroom acoustics, FM systems, or visual positioning) in order to disentangle relational support from technical and pedagogical adaptations.

### 4.3. Gender Differences in Health Outcomes

In accordance with Hypothesis 3, girls reported poorer well-being and higher levels of somatic and mental health complaints than boys across all groups. These gender differences were evident regardless of disability status and are consistent with previous research showing that girls tend to report higher levels of stress, somatic complaints, and internalizing symptoms during adolescence [23,24,53].

From an ecological standpoint, these patterns likely reflect a combination of individual-level factors (e.g., differences in stress sensitivity and coping strategies) and relational dynamics within the school microsystem, where girls may be more affected by relational stressors such as peer conflicts, social expectations, and perceived evaluation by others. These disparities appear to be particularly pronounced among adolescents with additional disabilities, suggesting an intersection between gender and disability-related vulnerability within broader social and educational contexts.

### 4.4. DHH Adolescents with Additional Disabilities as a Particularly Vulnerable Group

A key finding of the present study is that DHH adolescents with additional disabilities consistently reported the poorest outcomes across all measured domains of well-being, mental health, somatic complaints, and perceived teacher support. This group reported lower well-being, higher somatic complaints, higher mental health complaints, and lower perceived teacher support than all other adolescents, aligning with prior research showing heightened health risks among adolescents with multiple disabilities [42,44,45].

However, given the cross-sectional design, these differences should be interpreted as descriptive associations rather than causal effects. It is likely that the poorer outcomes observed among DHH adolescents with additional disabilities reflect a complex interplay of individual factors (e.g., functional limitations), familial resources, and broader educational and systemic conditions, rather than being attributable solely to the school environment.

From a practical perspective, this suggests that these adolescents may require more individualized and coordinated support strategies that address both educational and health-related needs. Longitudinal studies are needed to disentangle the directionality of these relationships and to better understand how these factors interact over time.

It should also be noted that factors such as socioeconomic status, differences in school resources, and access to specialized educational support were not directly measured in this study and may contribute to the observed disparities. Thus, the patterns observed likely reflect not only school-related factors but also broader structural inequalities and differential access to specialized support services—issues that should be addressed in future research.

## 5. Conclusions

This population-based study provides important insight into self-reported well-being, somatic complaints, mental health complaints, and perceived teacher support among deaf and hard-of-hearing (DHH) adolescents in mainstream schools. A key strength of the study is its reliance on adolescents’ own perspectives, revealing substantial disparities between hearing adolescents, DHH adolescents, and DHH adolescents with additional disabilities. Across all outcomes, adolescents with hearing loss, particularly those with additional disabilities, reported poorer health and well-being.

The findings underscore the critical role of the school environment during adolescence, a developmental period with long-term implications for mental health and functioning. Perceived teacher support emerged as a central contextual factor differentiating groups, suggesting that communication-sensitive teaching practices and increased awareness of hearing-related needs are essential for promoting inclusion and well-being in mainstream classrooms.

Overall, the study contributes contemporary population-based evidence to an area where empirical knowledge remains limited and highlights the need for coordinated educational and health-related support to promote equitable well-being among DHH adolescents in mainstream school settings.

### Implications for Practice and Policy

The findings underscore the central role of teachers in shaping the school experiences and well-being of DHH adolescents in mainstream settings. Given that perceived teacher support was lowest among DHH adolescents with additional disabilities, school-based efforts should prioritize strengthening teacher–student relationships and ensuring that teachers are equipped to recognize and respond to the diverse needs of these students.

From a pedagogical perspective, this may include increased professional development for teachers regarding hearing loss, communication barriers, and inclusive classroom practices, as well as strategies to foster supportive and responsive classroom climates. Although the present measure of teacher support captured general emotional, relational, and academic support, practice should also consider how communication-specific accommodations (e.g., classroom acoustics, FM systems, and visual positioning) can be integrated with relational support to promote both participation and well-being.

The results also highlight the importance of early identification of somatic and mental health complaints among DHH adolescents, such as persistent stress, fatigue, headaches, or social withdrawal. School-based interventions that combine teacher responsiveness, improved classroom communication, and coordinated educational and health-related support may help reduce health disparities and support positive developmental trajectories—particularly for DHH adolescents with additional disabilities.

More broadly, the study points to the need for multidisciplinary collaboration between schools, health services, and families to ensure equitable access to specialized support for DHH adolescents in mainstream school settings. Future studies should incorporate multilevel data to examine how classroom acoustics, use of assistive hearing technology, and teacher training in inclusive education shape DHH students’ experiences of support and well-being.

## 6. Limitations

Several limitations should be considered when interpreting the findings of this study. The cross-sectional design precludes conclusions about causality or developmental trajectories. Because of the cross-sectional design, we cannot determine whether poorer outcomes among DHH adolescents with additional disabilities are a cause or a consequence of their school experiences. Although clear associations were observed between well-being, somatic complaints, mental health complaints, and perceived teacher support, longitudinal studies are needed to examine how these relationships evolve over time. Consequently, we cannot determine the directionality of the observed associations, for example, whether lower perceived teacher support contributes to poorer well-being or whether adolescents with poorer well-being perceive their teachers as less supportive.

First, hearing loss was based on self-report and did not allow differentiation by degree of hearing loss or functional impact. This likely means that the DHH group is heterogeneous, and adolescents with more severe hearing loss or greater functional difficulties may have different experiences of well-being and school support than those with milder hearing loss. This limitation may have influenced the observed associations and should be considered when interpreting the findings.

Second, the study relied on adolescents’ self-reported data, which may be subject to reporting bias. However, the survey instruments have demonstrated acceptable reliability and have been applied repeatedly in population-based research with consistent results. Data collection was conducted anonymously during school hours, which may have influenced response patterns, as classroom-based surveys can be associated with inattentive responding. In addition, reliance on self-report for both predictors and outcomes may have introduced common-method bias and social desirability effects, as students’ perceptions of their health and of teacher support were collected using the same method and at the same time point. Future studies would benefit from incorporating multi-informant data (e.g., teacher or parent reports) or objective indicators where possible.

Second, the study relied on adolescents’ self-reported data, which may be subject to reporting bias. However, the survey instruments have demonstrated acceptable reliability and have been applied repeatedly in population-based research with consistent results. Data collection was conducted anonymously during school hours, which may have influenced response patterns, as classroom-based surveys can be associated with inattentive responding.

Third, different measures of well-being were used across age groups. While adolescents in grades 9 and upper-secondary school completed the WHO-5 Well-Being Index, a single-item measure of general well-being was used for younger adolescents in grade 7 to reduce respondent burden. Although this approach minimized missing data, future studies would benefit from using identical validated measures across age groups to enhance comparability.

Fourth, the study did not include information on several potentially important confounding factors, such as socioeconomic status, differences in school resources, or the specific types of educational and technical support provided to DHH students (e.g., access to special education services, hearing technology, or classroom accommodations). These unmeasured factors may have influenced both students’ health outcomes and their perceptions of teacher support and could partly explain the observed group differences. Future research would benefit from incorporating such contextual variables to better disentangle individual, familial, and school-level influences on well-being and health among DHH adolescents. The study did not include detailed school- or classroom-level variables such as class size, availability of specialized hearing support, teacher training in inclusive education, or school resources, which may influence students’ perceptions of teacher support and health outcomes.

Finally, although the study included a large population-based sample, the subgroup of DHH adolescents, particularly those with additional disabilities, was relatively small, which may have limited statistical power in some subgroup analyses.

## Figures and Tables

**Table 1:** Distribution of participants by grade, gender, and disability status (percentages in parentheses).

	7th Grade ^c^		9th Grade ^d^		Year 2 ^e^		Total
	Boys	Girls	Boys	Girls	Boys	Girls	
No disability	967 (18.1)	966 (18.1)	892 (16.7)	886 (16.6)	809 (15.1)	822 (15.4)	5342 (100)
DHH ^a^	37 (15.8)	35 (14.9)	31 (13.2)	36 (15.3)	61 (25.9)	35 (14.9)	235 (100)
DHH add ^b^	25 (11.3)	23 (10.4)	45 (20.4)	38 (17.2)	54 (24.4)	36 (16.3)	221 (100)
Total	1027 (17.7)	1026 (17.7)	968 (16.7)	960 (16.6)	924 (15.9)	893 (15.4)	5798 (100)

Note. ^a^ Deaf and hard-of-hearing. ^b^ DHH with additional disability. ^c^ 7th grade in compulsory schools. ^d^ 9th grade in compulsory schools. ^e^ Year 2 in upper-secondary schools. Totals reflect valid responses for gender, grade, and disability status.

**Table 2:** Means, standard deviations and Pearson correlations among the measured variables for DHH participants.

Variables	M	SD	1	2	3	4	5	6	7
1. WHO well-being ^a^	2.79	1.07	-						
2. General well-being	3.84	0.87	0.51	-					
3. Somatic problems	2.32	0.69	−0.51	−0.44	-				
4. Mental health complaints	2.79	0.83	−0.65	−0.53	0.55	-			
5. Perceived teacher *	3.88			0.74	0.26	−0.30	−0.22	−0.31	-

Note. ^a^ The 7th-grade students did not answer the WHO-5. Perceived teacher * = Perceived teacher support. *p* < 0.01 for all correlations.

**Table 3:** Means and standard deviations (shown in parentheses) for all variables by disability status and gender.

Disability	No disability			DHH ^a^			DHH additional ^b^		
Variables	Boys	Girls	Total	Boys	Girls	Total	Boys	Girls	Total
WHO well-being ^c^	3.41 (0.95)	2.62 (1.02)	3.01 (1.06)	2.95 (1.03)	2.59 (1.01)	2.80 (1.03)	2.58 (1.09)	2.09 (1.02)	2.37 (1.08)
General well-being	4.27 (0.76)	3.85 (0.89)	4.06 (0.85)	3.95 (0.88)	3.71 (0.83)	3.84 (0.87)	3.71 (0.98)	3.19 (1.08)	3.48 (1.05)
Somatic complaints	1.81 (0.57)	2.21 (0.65)	2.01 (0.64)	2.21 (0.73)	2.44 (0.62)	2.31 (0.69)	2.43 (0.79)	2.87 (0.73)	2.62 (0.79)
Mental complaints	2.17 (0.77)	2.94 (0.86)	2.55 (0.91)	2.56 (0.82)	3.06 (0.78)	2.79 (0.83)	2.71 (0.89)	3.50 (0.78)	3.06 (0.93)
Perceived teacher support	4.10 (0.76)	3.93 (0.73)	4.01 (0.75)	4.05 (0.73)	3.71 (0.70)	3.89 (0.74)	3.67 (0.97)	3.63 (0.83)	3.66 (0.91)

Note. ^a^ Deaf and hard of hearing. ^b^ DHH with additional disabilities. ^c^ Participants in the 7th grade did not answer the WHO-5.

**Table 4:** Means and standard deviations (shown in parentheses) for well-being, somatic complaints and mental health complaints by year in school, disability status and gender.

7th grade ^a^	No disability		DHH ^e^		DHH additional ^f^	
	Boys	Girls	Boys	Girls	Boys	Girls
General well-being ^b^	4.33 (0.71)	3.99 (0.91)	4.23 (0.65)	3.65 (0.98)	3.88 (0.88)	3.22 (1.17)
Somatic complaints	1.76 (0.54)	2.05 (0.65)	1.99 (0.61)	2.39 (0.64)	2.21 (0.67)	2.91 (0.55)
Mental health complaints	1.98 (0.70)	2.62 (0.89)	2.18 (0.69)	2.93 (0.87)	2.39 (0.75)	3.45 (0.72)
9th grade ^c^						
					
WHO well-being	3.51 (0.93)	2.66 (1.04)	3.03 (1.00)	2.34 (0.98)	2.51 (1.07)	1.81 (0.85)
Somatic complaints	1.79 (0.56)	2.24 (0.61)	2.29 (0.66)	2.61 (0.58)	2.42 (0.75)	2.89 (0.74)
Mental health complaints	2.19 (0.77)	3.06 (0.81)	2.75 (0.78)	3.30 (0.67)	2.91 (0.87)	3.52 (0.72)
Year 2 ^d^						
					
WHO well-being	3.31 (0.96)	2.58 (0.99)	2.91 (1.05)	2.86 (0.99)	2.64 (1.11)	2.38 (1.11)
Somatic complaints	1.88 (0.60)	2.37 (0.64)	2.29 (0.80)	2.32 (0.61)	2.54 (0.86)	2.82 (0.83)
Mental health complaints	2.37 (0.79)	3.19 (0.77)	2.68 (0.84)	2.95 (0.75)	2.70 (0.93)	3.51 (0.89)

Note. ^a^ 7th grade in compulsory schools. ^b^ Participants in 7th grade did not answer the WHO questions, so general well-being is used here. ^c^ 9th grade in compulsory schools. ^d^ Year 2 in upper secondary schools. ^e^ Deaf and hard -of -hearing. ^f^ DHH with additional disabilities.

## Data Availability

The original contributions presented in this study are included in the article. Further inquiries can be directed to the corresponding author.
